# Delivery of a Chlamydial Adhesin N-PmpC Subunit Vaccine to the Ocular Mucosa Using Particulate Carriers

**DOI:** 10.1371/journal.pone.0144380

**Published:** 2015-12-11

**Authors:** Aleksandra Inic-Kanada, Marijana Stojanovic, Simone Schlacher, Elisabeth Stein, Sandra Belij-Rammerstorfer, Emilija Marinkovic, Ivana Lukic, Jacqueline Montanaro, Nadine Schuerer, Nora Bintner, Vesna Kovacevic-Jovanovic, Ognjen Krnjaja, Ulrike Beate Mayr, Werner Lubitz, Talin Barisani-Asenbauer

**Affiliations:** 1 OCUVAC–Center of Ocular Inflammation and Infection, Laura Bassi Centers of Expertise, Center for Pathophysiology, Infectiology and Immunology, Medical University of Vienna, Vienna, Austria; 2 Department of Research and Development, Institute of Virology, Vaccines and Sera–TORLAK, Belgrade, Serbia; 3 BIRD-C GmbH&CoKG, Vienna, Austria; Midwestern University, UNITED STATES

## Abstract

Trachoma, caused by the intracellular bacterium *Chlamydia trachomatis* (Ct), remains the world’s leading preventable infectious cause of blindness. Recent attempts to develop effective vaccines rely on modified chlamydial antigen delivery platforms. As the mechanisms engaged in the pathology of the disease are not fully understood, designing a subunit vaccine specific to chlamydial antigens could improve safety for human use. We propose the delivery of chlamydia-specific antigens to the ocular mucosa using particulate carriers, bacterial ghosts (BGs). We therefore characterized humoral and cellular immune responses after conjunctival and subcutaneous immunization with a N-terminal portion (amino acid 1–893) of the chlamydial polymorphic membrane protein C (PmpC) of Ct serovar B, expressed in probiotic *Escherichia coli* Nissle 1917 bacterial ghosts (EcN BGs) in BALB/c mice. Three immunizations were performed at two-week intervals, and the immune responses were evaluated two weeks after the final immunization in mice. In a guinea pig model of ocular infection animals were immunized in the same manner as the mice, and protection against challenge was assessed two weeks after the last immunization. N-PmpC was successfully expressed within BGs and delivery to the ocular mucosa was well tolerated without signs of inflammation. N-PmpC-specific mucosal IgA levels in tears yielded significantly increased levels in the group immunized via the conjunctiva compared with the subcutaneously immunized mice. Immunization with N-PmpC EcN BGs via both immunization routes prompted the establishment of an N-PmpC-specific IFNγ immune response. Immunization via the conjunctiva resulted in a decrease in intensity of the transitional inflammatory reaction in conjunctiva of challenged guinea pigs compared with subcutaneously and non-immunized animals. The delivery of the chlamydial subunit vaccine to the ocular mucosa using a particulate carrier, such as BGs, induced both humoral and cellular immune responses. Further investigations are needed to improve the immunization scheme and dosage.

## Introduction

Trachoma is the most common cause of preventable blindness in underdeveloped countries. Ocular disease is primarily caused by an acute inflammatory response elicited by the infection of the host cell and the T cell response to *Chlamydia trachomatis* (Ct). However, infections resolve through both antibody- and Th1-mediated mechanisms [1–8]. Currently, no vaccines for the disease are available for humans; however, intensive efforts to develop a trachoma vaccine including human trials, date back to the 1960s [9–15].

The delivery of vaccines via the ocular conjunctiva may be an attractive option for mucosal immunization against ocular pathogens as it could induce a first line of defense at the ocular surface against several disorders that cause blindness (e.g., trachoma, herpes corneae, and acanthamoeba keratitis). Ocular mucosa possesses features for generating a specific immune response in the conjunctiva-associated lymphoid tissue (CALT). CALT is assumed to play a key role in protection of the ocular surface by initiating and regulating immune responses [16].

In our previous work, we demonstrated that conjunctival delivery of tetanus toxoid induced high local mucosal IgA production and a local Th1-driven immune response when mixed with a particulate adjuvant [17]. For further ocular vaccine development, we seek a particulate carrier that is i) readily taken up by ocular surface cells, ii) safe for the conjunctival route of immunization, iii) non-living and iv) able to carry foreign subunit antigens.

The use of bacterial ghosts (BGs) as a vaccine carrier to elicit an immune response using a wide range of immunization routes and animal models was investigated in previous studies [18–23]. BGs are non-living, Gram-negative bacterial cell envelopes that are devoid of their cytoplasmic contents yet maintain their cellular morphology, antigenicity and immune-stimulating compounds. BGs are particles that contain a surface with various structures involved in antigen recognition and uptake, and are therefore readily recognizable by antigen presenting cells [24]. BGs are similar to naturally engineered liposomes with two membranes separated by a periplasmic space where the rigid peptidoglycan corset and membrane-derived oligosaccharides are located. In recombinant BGs, foreign proteins can be anchored in different membrane compartments prior to *E*-mediated lysis, e.g. in the inner membrane via specific membrane anchor sequences or transported to the periplasmic space via specific leader sequences [25–27]. Furthermore, the BGs platform can express antigens (e.g., membrane proteins) that are difficult to produce in large quantities needed for vaccine development.

For ocular mucosal delivery, we demonstrated that the BGs were readily taken up and well tolerated by human conjunctival epithelial cells (CECs) *in vitro* and guinea pig CECs *in vivo* [28]. We also confirmed that BGs preserve the outer membrane structures of parental Gram-negative bacteria, which is an important feature for their uptake by innate immune cells and can also express chlamydial-specific subunit antigens [29].

The aim of this study was first to evaluate the tolerability of conjunctival immunization using a subunit antigen (N-terminal portion of chlamydial polymorphic membrane protein C; N-PmpC) delivery by BGs produced from *E*. *coli* Nissle1917 (EcN) as well as the capability of particulate N-PmpC-containing EcN BGs to initiate an antigen-specific immune response after immunization via the ocular mucosa in BALB/c mice. The chlamydial adhesin PmpC was selected because it is expressed in both reticulate and elementary bodies during the bi-phasic developmental cycle of *Chlamydiae* [30–32]. An additional incentive for PmpC selection was that this adhesin is involved in early stage chlamydia-host cell interactions via its N-terminal portion [33], and it can adhere to human cells [34]. Recently, the antibody response in trachoma patients was investigated in a genome-wide scale [35]. It was found that PmpC was significantly associated with trichiasis but it remained unknown whether PmpC and immune responses induced could mechanistically contribute to ocular pathology during Ct infection. Epitope mapping of PmpC_amino acid (aa) 605–840_ demonstrated broad B cell recognition and the full length protein has been shown to react with sera from infected minipigs [36].

Mice do not develop ocular diseases after infection with *Chlamydiae*; therefore, we also evaluated the *in vivo* protection after immunization with N-PmpC EcN BGs in the guinea pig inclusion conjunctivitis model.

## Materials and Methods

### Ethics statement

All experiments were approved by the "Ethics Committee for the Welfare of Experimental Animals" and by the committee section at the Institute of Virology, Vaccines and Sera–TORLAK. All experiments conformed to the Serbian laws and European regulations on animal welfare (Approval No. 011-00-00510/2011-05/2A). Every effort was made to minimize animal suffering. Mice that were immunized were anesthetized by intraperitoneal (i.p.) administration of a mixture of xylazine (Sigma-Aldrich, Kansas, KS, USA) and ketamine (Richter Pharma AG, Wels, Austria). The method for mice euthanasia was cervical dislocation. Guinea pigs, immunized and challenged, were observed daily by trained animal care staff, and animals requiring care were referred to the attending veterinarian for immediate care. Terminal euthanasia was carried out by lethal CO_2_ overdose. We did not observe any unexpected deaths of animals during this study.

### Antigens

EcN BGs were produced from probiotic EcN strain by the controlled expression of the phage-derived lysis gene *E*, as described previously [26, 37]. Recombinant N-PmpC EcN BGs were prepared from strain EcN (pBGKB-N-PmpC) carrying the N-PmpC sequence (2679 bp, encoding for PmpC_aa 1–893_ portion) from Ct, serovar B (ATCC® VR-573™). Plasmid pBGKB is a kanamycin resistant version of plasmid pBAD/gIII B (Life Technologies, Carlsbad, CA, USA). The sequence of N-PmpC was cloned in frame to the leader peptide gIII, which directs the recombinant protein into the periplasmic space, and to a myc tag on the C-terminal end. Expression of N-PmpC is tightly regulated by the *araBAD* promoter. As a control empty EcN BGs were produced from strain EcN (pBGKB). Both EcN strains were transformed with lysis plasmid pGLysivb carrying the lysis gene *E* from bacteriophage PhiX174 under control of a temperature inducible promoter/repressor system and a gentamycin resistance cassette [37]. Bacteria were grown in animal proteins-free LB medium supplemented with gentamycine (10 μg/ml) and kanamycin (50 μg/ml). Within the exponential growth phase recombinant protein expression was induced by addition of L-arabinose (0.1%) followed by the *E*-lysis induction via temperature upshift from 35°C to 42°C. Inactivation was carried out with β-propiolactone. After washing with deionised water, the BGs were lyophilized, weighed and stored at room temperature for 12 month prior to use. All BGs samples were reconstituted in PBS prior further use.

### Quantification of N-PmpC in N-PmpC EcN BGs

N-PmpC_aa1-893_ expression was quantified using Western blot analysis. BGs expressing major outer membrane protein (MOMP) were used as standards. Briefly, 10 mg of lyophilized BGs with expressed MOMP were suspended in 1000 μl of dH_2_O (conc. 10 μg/μl). A 200 μl volume was removed, spun down, resuspended and diluted with NuPage Sample Buffer (reduced; Life Technologies, Carlsbad, CA, USA) and heated for 10 min at 99°C. The amounts of MOMP loaded per lane was as follows: 560 ng standard 1; 760 ng standard 2; 1200 ng standard 3; 1600 ng standard 4. Lyophilized N-PmpC EcN BGs were diluted in loading buffer (reduced; Life Technologies, Carlsbad, CA, USA). A total of 10 mg lyophilized N-PmpC EcN BGs was resuspended in 1000 μl dH_2_O (10 μg/μl). The amount loaded per lane was 10 μg of N-PmpC BGs. The membrane was developed using an anti-myc-HRP antibody. Quantification of N-PmpC was performed with QuantityOne Software using the ChemiDocXRS software (Bio-Rad, Hercules, CA, USA).

### Animals

Ten-week-old BALB/c female mice and six-week-old Hartley strain (300–350 g) female guinea pigs were housed at the Animal Facility of the Institut TORLAK, at a temperature of 21°C with 12 h light/dark cycles and water and food *ad libitum*. The mice were weighed before and during the first 15 days of immunization.

### Immunization schedules

The study was conducted by conjunctivally (10 μl per mouse containing 50 μg of N-PmpC EcN BGs or EcN BGs) or subcutaneously (100 μl containing 500 μg/ml of N-PmpC EcN BGs or EcN BGs) immunizing BALB/c mice (six groups) on three separate occasions at two-week intervals. Unless stated otherwise, each experimental group routinely consisted of ten animals. Mice were immunized on days 0, 14, and 28 via either the conjunctival (conj//) or subcutaneous (s.c.//) route. Two weeks after the final immunization, local and systemic immune responses were evaluated. Age-matched, non-immunized mice were used as controls. Antigens were applied onto the conjunctival sacs using a micropipette. The mice were maintained under anesthesia for 30 minutes to prevent removal of the immunization solution. Subcutaneously immunized mice were treated the same way. Guinea pigs used for the protection assay were immunized using the same immunization scheme and the same antigen concentration as described above.

### Sample collection

Tear-wash samples were obtained by lavage with 10 μl of phosphate-buffered saline (PBS) per mouse eye. The collected tears were supplemented with a protease inhibitor cocktail (Thermo Scientific, USA) and stored at -80°C.

Blood sera samples were collected by bleeding from the retro-orbital sinuses two weeks after the completion of the immunization protocol. The collected sera were complement depleted, aliquoted and stored at -20°C. Blood sera samples and spleens were collected on the day of sacrifice (two weeks after the third immunization).

### Detection of N-PmpC EcN BG-specific IgG and IgA in mouse sera and tears

Enzyme-linked immunosorbent assay (ELISA) plates (MaxiSorp; Nunc, Roskilde, Denmark) were coated (50 μl/well) with the recombinantly produced 893 amino acids long N-terminal portion of PmpC (10 μl/ml in PBS) and incubated for 1 h at 37°C. A solution of 1% (w/v) BSA/PBS was applied as a blocking reagent for 2 h at room temperature (RT). This blocking step and all subsequent ELISA steps were followed by washes with 0.05% (v/v) Tween 20 in PBS (four times, 200 μl/well). Appropriately diluted serum (1:100) and tear wash (1:10) samples were incubated overnight at 4°C. Antigen-specific antibody binding was detected after 1 h incubation at RT using biotin-labeled anti-mouse IgG (Sigma, Steinheim, Germany) and biotin-labeled anti-mouse IgA antibody (BioLegend, San Diego, CA, USA). Antigen-antibody interactions were visualized using the extrAvidin-peroxidase/o-phenylendiamine system (Sigma, Steinheim, Germany), and absorbances were recorded at 492/620 nm (A_492/620_). The cutoff value for each system was defined according to the A_492/620_ value obtained from the “negative control” wells [1% BSA (w/v) in PBS] plus 3 × standard deviation (SD).

### Isolation of splenocytes

Spleens from immunized and control mice were aseptically isolated, trimmed of all excess tissue and placed in 5 ml of sterile complete RPMI 1640 (Sigma-Aldrich) supplemented with inactivated 10% fetal calf serum (FCS). Lymphocytes were harvested in 5% FCS/RPMI 1640 and passed through sterile nylon cell strainers (70 μm, BD Bioscience) to remove large particles. Cell suspensions were centrifuged at 300 × g (10 min) (SIGMA 3K18, Sigma Laboratory Centrifuges GmbH). Suspensions were erythrocyte-depleted by short incubation (15 s) in sterile redistilled water (3 ml) before the addition of 0.3 M NaCl/5% FCS/RPMI 1640 (3 ml). After centrifugation, the lymphocytes were washed thrice in 10% FCS/RPMI 1640 with centrifugation at 300 × g (5 min). The cells were finally diluted in 10% FCS/50 μM β-mercaptoethanol/RPMI 1640 to a concentration of 2 × 10^6^ cells/ml. The viability of these cell preparations, as determined by trypan blue exclusion, was greater than 95%.

### Cell proliferation assay

Splenocytes were plated into 96-well plates (100 μl/well, 2 × 10^6^ cell/ml in 10% FCS/50 μM β-mercaptoethanol/RPMI 1640) and incubated for 48 h (5% CO2, 37°C) without additional stimulation or in the presence of the stimulator N-PmpC (10 μg/ml). Cell Counting *Kit-*8 reagent (10 μl/well, Sigma Aldrich) was added, and the cells were incubated at 37°C and 5% CO_2_ for 4 h. Reactions were stopped by the addition of 1% (w/v) sodium dodecyl sulfate (SDS; 10 μl/well), and absorbance values were measured at 450/650 nm (A_450/650_) using a spectrophotometer (Ascent 6–384 [Suomi], MTX Lab Systems Inc., Vienna, VA, USA). The number of viable cells per well (NVC) was calculated using a standard curve of the number of cells plotted against A_450/650_. Discrete pools of non-stimulated cells were used as standards after counting in the presence of trypan blue (Countess Automated Cell Counter, Invitrogen). Standard suspensions were plated in serial dilutions prior to centrifugation and further treated identically as the experimental wells that received the stimulator. A proliferation index (PI) for each stimulated cell suspension was calculated per individual animal. The PI index was defined as the ratio of NVC present in stimulated (S) samples to NVC present in non-stimulated (nS) samples, such that PI = NVCS:NVCnS.

### Cytokine analysis

Splenocytes (2 × 10^6^ cells/ml) were plated in 24-well and incubated for 48 h (5% CO2, 37°C) without additional stimulation or in the presence of the stimulator N-PmpC (10 μg/ml). The production of interferon gamma (IFNγ) and interleukin-4 (IL-4) was analyzed by measuring the supernatant concentrations of non-stimulated and N-PmpC-stimulated splenocyte cultures using sandwich ELISAs with commercially available monoclonal antibodies (eBioscience, Vienna, Austria). Unlabeled monoclonal antibodies specific for IFNγ (1 μg/ml) and IL-4 (2 μg/ml) were coated onto microtiter plates (MaxiSorp, Nunc) by overnight adsorption at 4°C, and 1% (w/v) BSA/PBS was used to block the plates (2 h) at RT. Blocking and all subsequent ELISA steps were followed by a wash step with 0.05% (v/v) Tween 20 in PBS (four times, 200 μl/well). Biotin-labeled antibodies specific for IFNγ (2 μg/ml) and IL-4 (1 μg/ml) were diluted in 1% BSA/PBS, added to the wells and incubated for 1h at RT. The extrAvidin-alkaline phosphatase/p-nitrophenyl phosphate system (Sigma-Aldrich) was used to visualize antigen–Ab interactions. Absorbance was monitored at 405 nm (A_405_). Standard curves were created using commercially available recombinant mouse IL-4 and IFNγ standards (eBioscience, Vienna, Austria).

### Acute tolerance assay

With histopathology examination of guinea pig conjunctiva our primary goal was to check if the immunization with BGs (bio-particles) causes any acute effect on the conjunctiva immediately after immunization. This was investigated macroscopically and microscopically and is in accordance with Organization for Economic Cooperation and Development 405 (OECD 405) guidelines for the testing of chemicals (*in vivo*) test for acute eye irritation/corrosion. Five guinea pigs per group (group labeled as 2 h and group labeled as 24 h) received 25 μl containing 50 μg of N-PmpC-EcN BGs. One guinea pig (per time point) was used as a sham-treated control. Two and 24 h after the instillation, animal discomfort, and clinical signs in the conjunctiva, corneas, and lids were evaluated by an experienced ophthalmologist for possible acute toxicity of the N-PmpC EcN BGs. Conjunctival tissue was removed two and 24 h after immunization and fixed in 1% formaldehyde in PHEM buffer (60 mM PIPES, 25 mM HEPES, 10 Mm EGTA, and 2 mM MgCl_2_). Paraffin embedded sections were processed for histology. The sections (4 μm) were routinely stained with hematoxylin and eosin. For the comparison between an inflamed or infiltrated section vs. normal section, we used data from the literature [38–41].

### *Chlamydia caviae* conjunctival infections

*Chlamydia caviae* has been kindly provided by Prof. Roger G. Rank. The organism stocks were prepared according to standard methodology in McCoy cells [42] and frozen at -80°C in sucrose-phosphate-glutamate (SPG) buffer until needed. Guinea pigs were anesthetized with a mixture of ketamine (30 mg/kg) and xylazine (2 mg/kg) applied intramuscularly. Infection was performed on anesthetized guinea pigs by instilling 25 μl of SPG buffer containing 1 × 10^6^ IFUs of *C*. *caviae* directly into the conjunctival sac with a micropipette. The control group of animals received SPG buffer only.

### Pathology scoring

An experienced ophthalmologist who was blinded to the experimental groups observed and scored the eyes of guinea pigs in a protection assay on a daily basis [43, 44]. In brief, the palpebral and the bulbar conjunctiva were evaluated for erythema, edema, and exudation in each animal. Each observation was classified into 5 categories: (0.5) trace pathologic response, (1) slight erythema or edema of either the palpebral or the bulbar conjunctiva, (2) definite erythema or edema of either the palpebral or the bulbar conjunctiva, (3) definite erythema or edema of both the palpebral and the bulbar conjunctiva, or (4) definite erythema or edema of both the palpebral and the bulbar conjunctiva plus the presence of exudate.

### Statistical analysis

The statistical significance of the observed differences was evaluated using 1-way repeated measures analysis of variance (ANOVA). A probability (p) value of 0.05 was set as a limit of significance. The correlation between variables was evaluated by Pearson’s bivariate correlation analysis. All statistical analyses were performed by software: IBM SPSS Statistics 20.

## Results

### Recombinant EcN BGs carrying the N-PmpC were successfully produced

**Fig 1:**
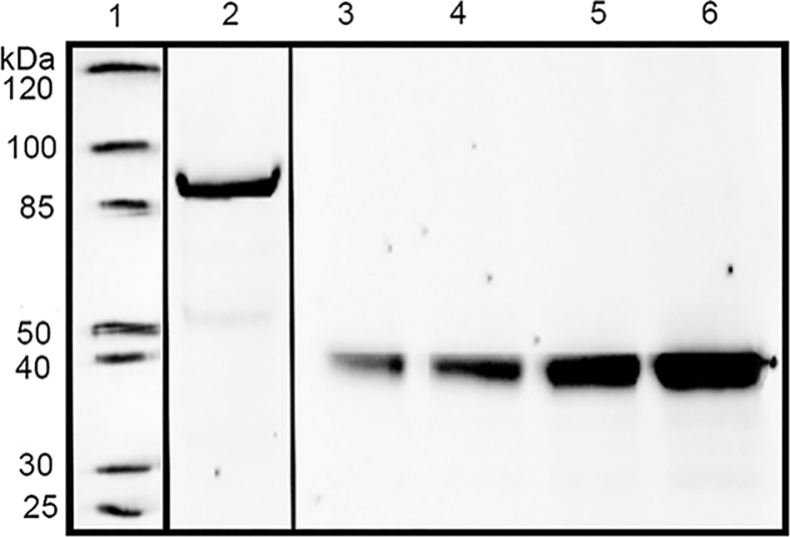
The expression of the N-PmpC was quantified using Western blot. BGs expressing major outer membrane protein (MOMP) were used as standards. Line 1, molecular weight markers; line 2, N-PmpC expressed within EcN BGs (MW 92.1 kD), line 3, 560ng MOMP standard 1; line 4, 760ng MOMP standard 2; line 5, 1200ng MOMP standard 3; line 6, 1600ng MOMP standard 4. The membrane was developed using anti-myc-HRP antibody. Quantification of the N-PmpC was performed with the QuantityOne Software in the ChemiDocXRS program.

BGs were produced in 20 L working volume, lyophilized in aliquots and stored at RT until further use. Western blot analysis of the N-PmpC antigen expression resulted in a clear band at the approximate weight of the N-PmpC polypeptide (92,1 kDa; Fig 1). N-PmpC EcN BGs were calculated to contain ~230 ng N-PmpC per 1 μg BG. The immunization dose for N-PmpC EcN BGs was 50 μg/animal, which is equivalent to ~11 μg N-PmpC per dose.

### N-PmpC-specific mucosal IgA levels in BALB/c mice tears are induced locally after conjunctival immunization with N-PmpC EcN BGs

**Fig 2:**
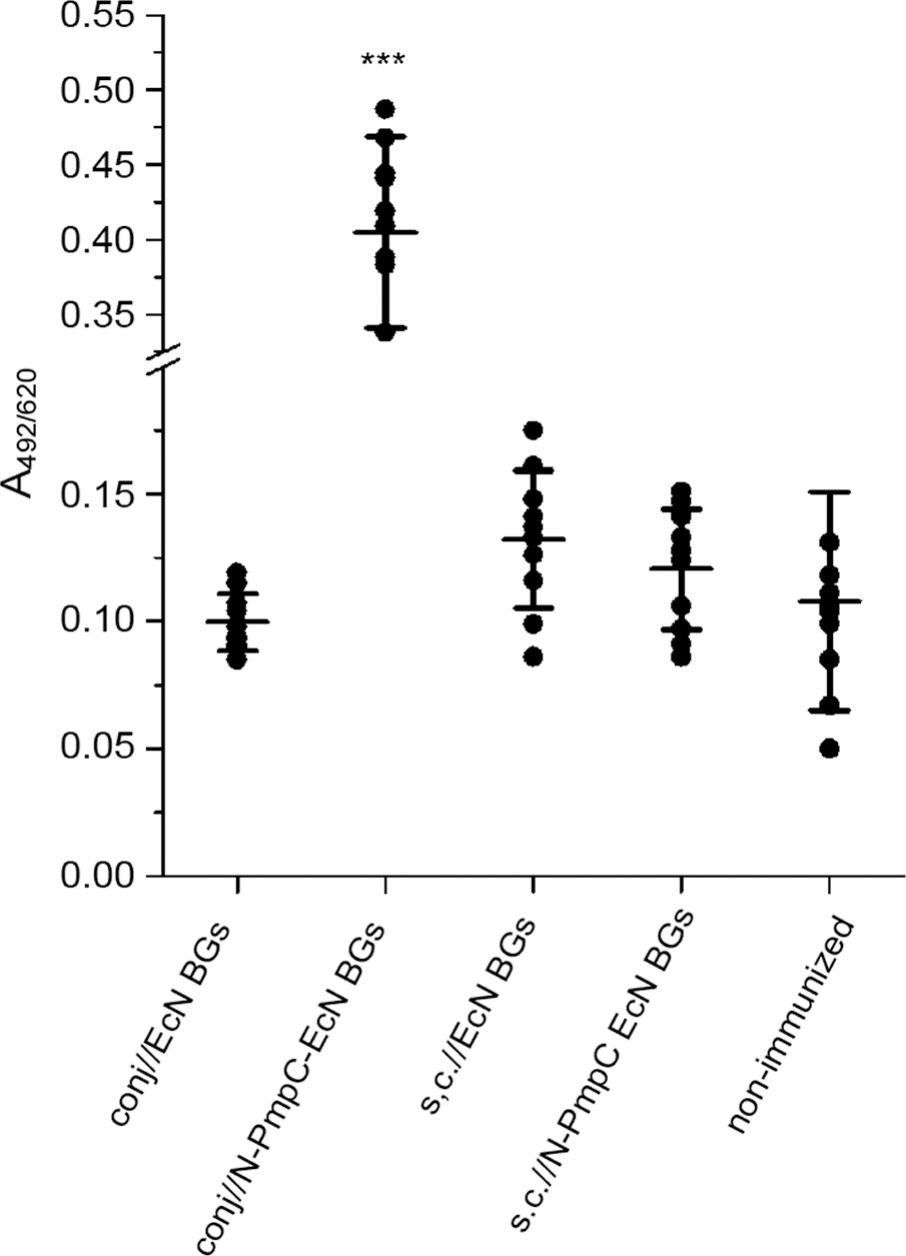
Levels of anti-N-PmpC mucosal IgA in tear washes from BALB/c mice obtained by two routes of immunization. Samples were collected two weeks after the completion of the immunization schedule and were assayed by ELISA (dilution 1:10). Each dot represents one sample. Lines indicate the mean values [A_492/620_ ± SD (n = 10)] calculated for each group. The statistical significance of the observed differences was evaluated using 1-way repeated ANOVA using non-immunized group as a reference (*p <0*.05*, *p <0*.005**, *p <0*.0001***).

**Fig 3:**
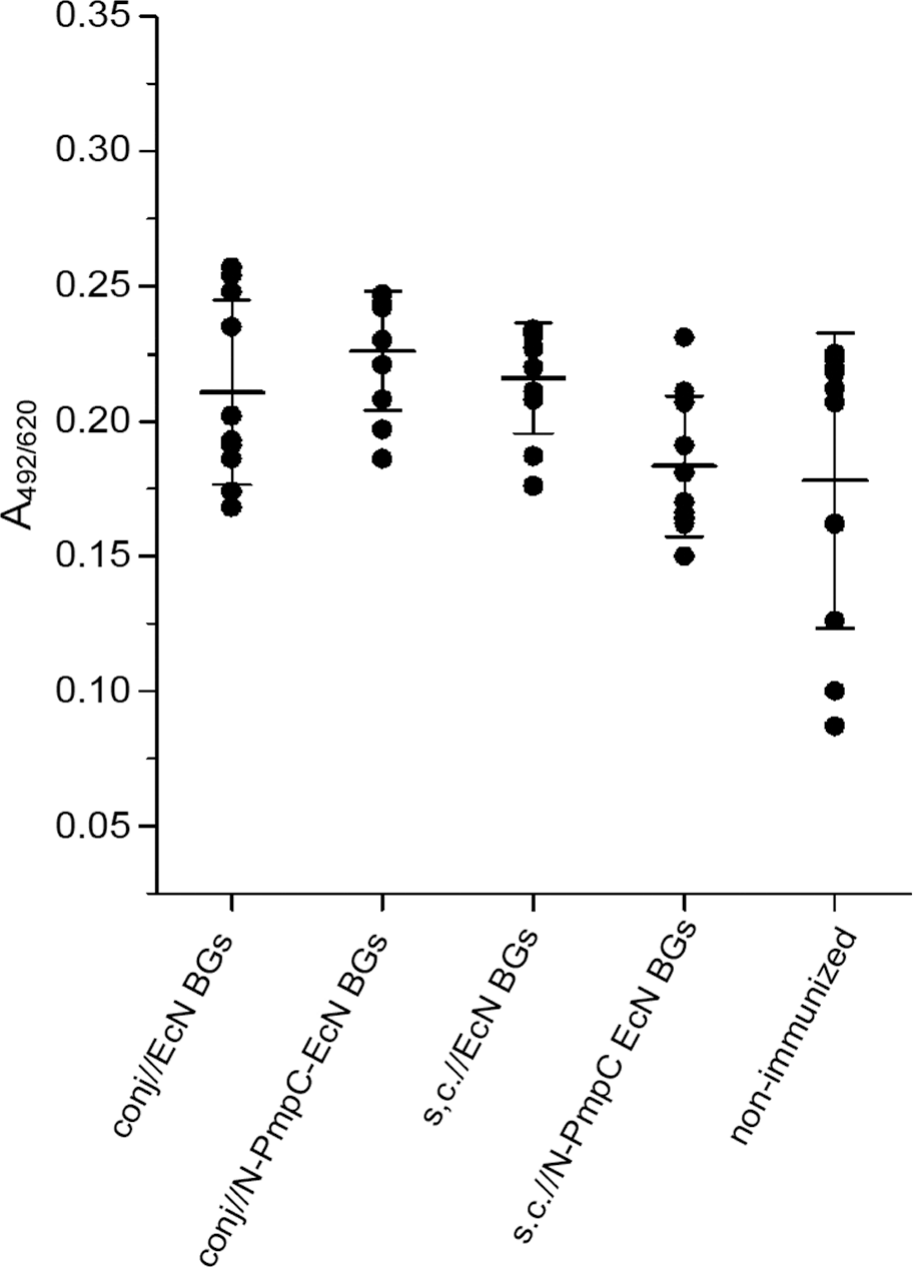
Levels of N-PmpC-specific IgA in the sera of BALB/c mice immunized via conjunctiva and subcutaneously. Serum samples were collected two weeks after the completion of the immunizations and were assayed by ELISA (dilution 1:100). Each dot represents one sample. Lines indicate the mean values [A_492/620_ ± SD (n = 10)] calculated for each group.

Immunization via the conjunctiva resulted in significantly increased N-PmpC-specific mucosal IgA levels compared with those found in tears from non-immunized control mice as well as subcutaneously immunized mice (in both cases p < 0.0001). Mucosal IgA values in mice that were subcutaneously immunized with N-PmpC EcN BGs or with EcN BGs (subcutaneously and via the conjunctiva) were comparable to those observed in control mice (Fig 2). In addition, N-PmpC-specific serum IgA levels recorded for sera of N-PmpC EcN BGs conjunctivally and subcutaneously immunized mice were comparable to the levels recorded for the non-immunized group (Fig 3).

### Subcutaneous immunization with N-PmpC EcN BGs elicited high levels of antigen-specific IgG in tears and sera

**Fig 4:**
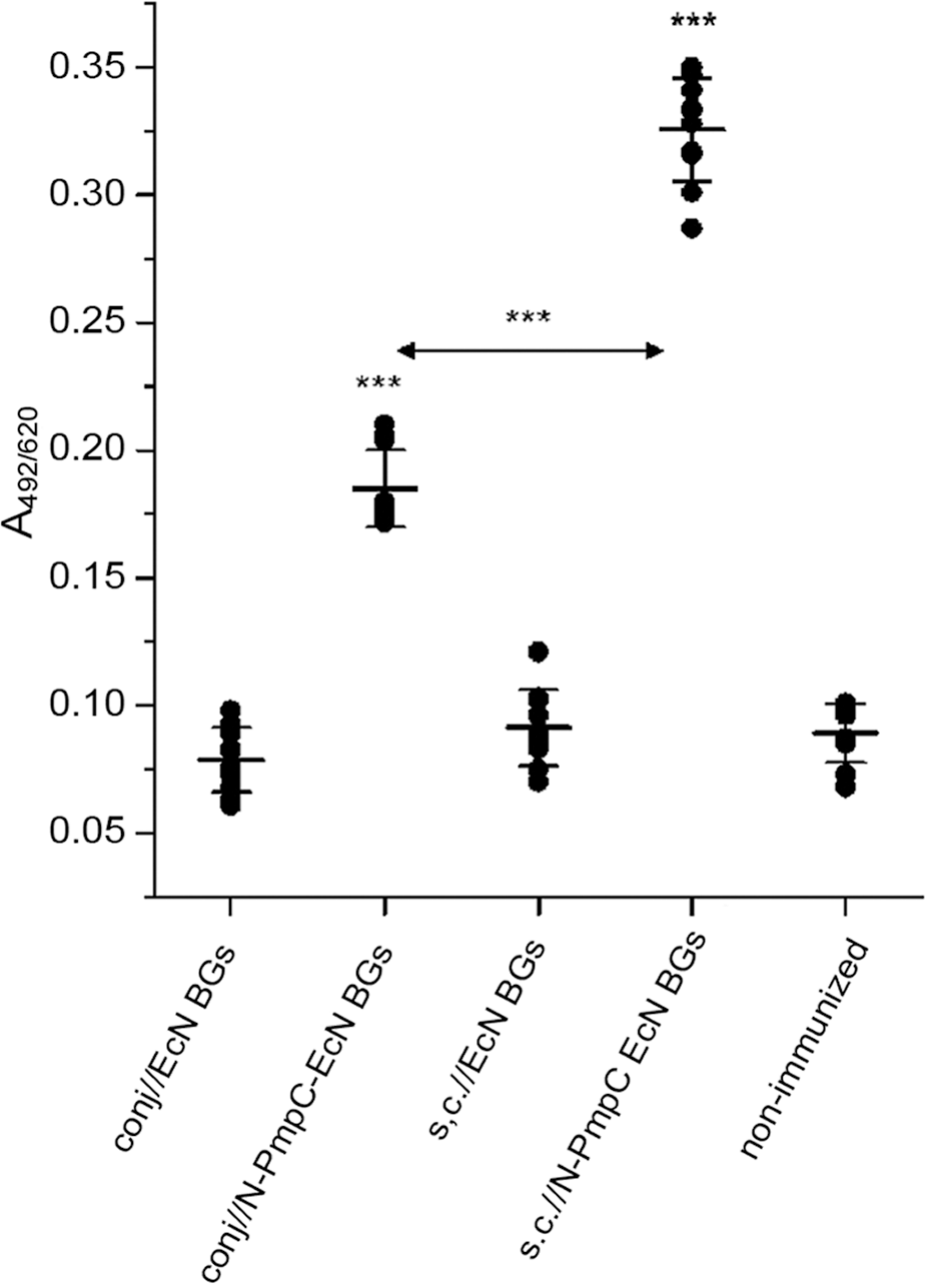
Levels of N-PmpC-specific IgG in tear washes from BALB/c mice that were immunized according to the assigned protocols. Samples were collected two weeks after the completion of the immunizations and were assayed by ELISA (dilution 1:10). Each dot represents one sample. Lines indicate the mean values [A_492/620_ ± SD (n = 10)] calculated for each group. The statistical significance of the observed differences was evaluated using 1-way repeated ANOVA (*p <0*.05*, *p <0*.005**, *p <0*.0001***). Non-immunized group was used as a reference unless otherwise indicated by two head-arrow.

**Fig 5:**
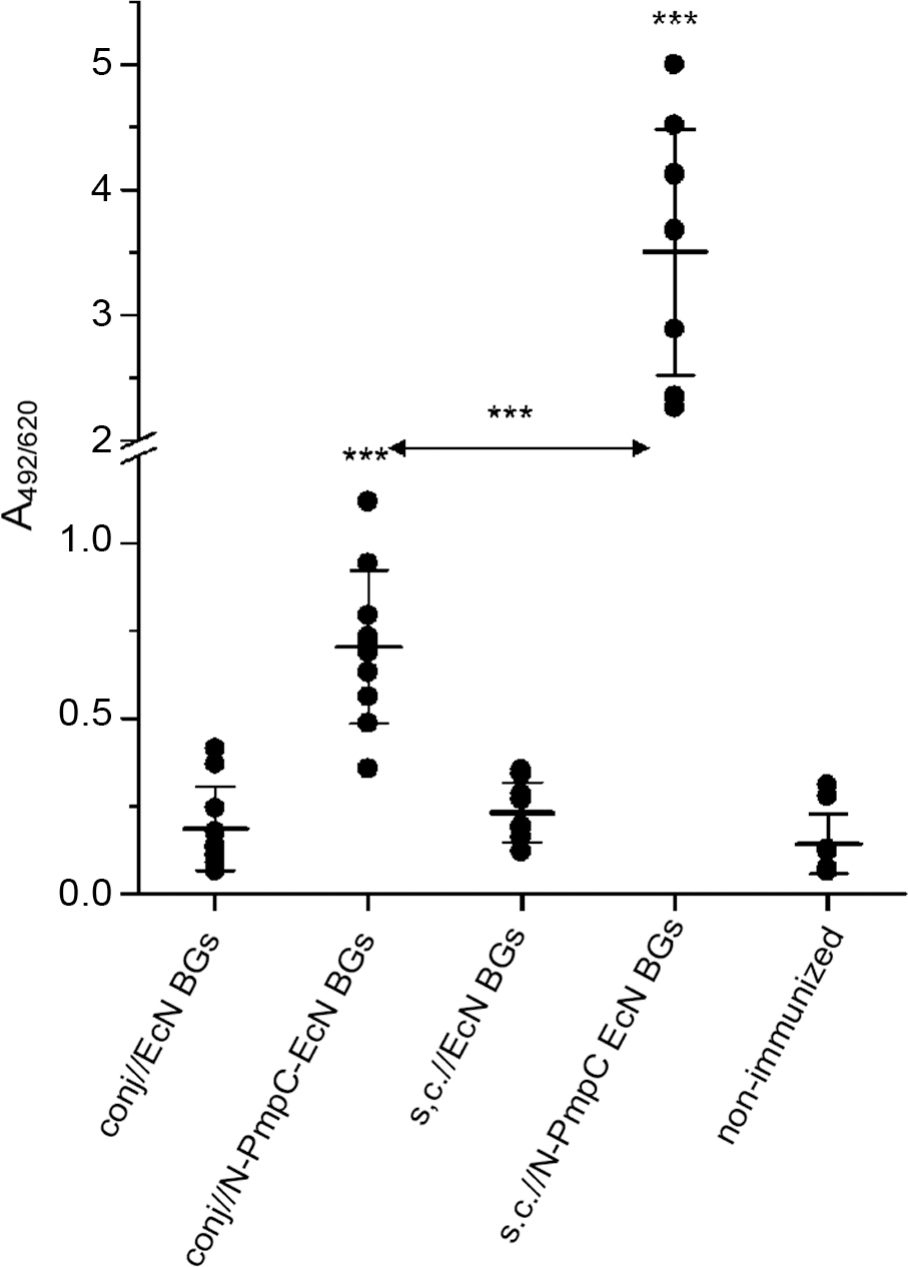
Levels of N-PmpC-specific IgG in the sera of BALB/c mice immunized via the conjunctiva or subcutaneously. Serum samples were collected two weeks after the completion of the immunizations and were assayed by ELISA (dilution 1:100). Each dot represents one sample. Lines indicate the mean values [A_492/620_ ± SD (n = 10)] calculated for each group. The statistical significance of the observed differences was evaluated using 1-way repeated ANOVA (*p <0*.05*, *p <0*.005**, *p <0*.0001***). In all cases when the non-immunized group was not a reference, compared groups are indicated by arrows.

BALB/c mice immunized subcutaneously with N-PmpC EcN BGs elicited significantly higher (p < 0.0001) levels of N-PmpC-specific IgG in tears compared to conjunctival immunization (Fig 4). N-PmpC-specific IgG levels in both immunization routes were significantly increased compared to non-immunized controls (in all cases p < 0.0001). Subcutaneous immunization with N-PmpC EcN BGs also resulted in significantly higher systemic production of N-PmpC-specific IgG compared to conjunctivally immunized mice (p < 0.0001) (Fig 5). A significant positive correlation (p = 0.0089, *Pcc* = 0.89) between N-PmpC-specific IgG levels in tears and serum was obtained. Immunization with EcN BGs either subcutaneously or via the conjunctiva did not affect levels of N-PmpC-specific IgG in serum and tears (p > 0.05 in comparison to the non-immunized control group).

### BALB/c mice splenocytes proliferated in response to N-PmpC

**Fig 6:**
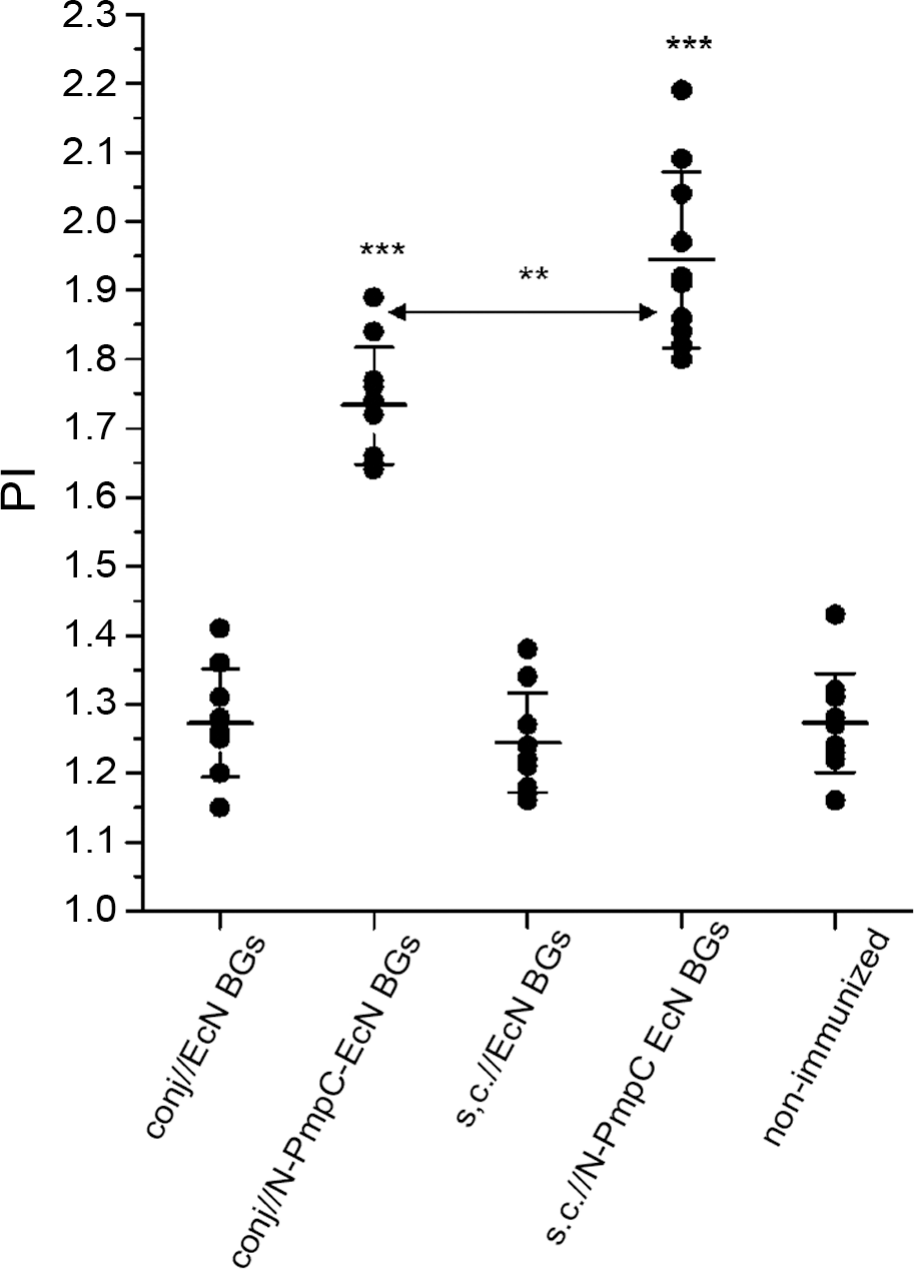
The proliferation indices (PI) of N-PmpC-stimulated splenocytes from BALB/c mice immunized according to the assigned immunization protocol. The numbers of viable splenocytes were assessed by *Cell* Counting Kit-*8* assay following a 48 h cultivation in 10% FCS/50 μM β-mercaptoethanol/RPMI 1640 medium supplemented or not with N-PmpC (10 μg/ml). Each dot represents PIs calculated for individual mouse. Lines indicate the mean values [A_492/620_ ± SD (n = 10)] calculated for each group. The statistical significance of the observed differences in PIs between groups treated according to the assigned protocols was evaluated using 1-way repeated ANOVA. *(p < 0*.05*, *p < 0*.005**, *p <0*.0001***). Non-immunized group was used as a reference unless otherwise indicated by two head-arrow.

Splenocytes from N-PmpC EcN BGs-immunized mice were assessed for their ability to proliferate in response to *in vitro* stimulation by N-PmpC. Their proliferative responses to N-PmpC were compared to splenocytes’ responses obtained from non-immunized. Following immunization with N-PmpC EcN BGs via the conjunctiva and subcutaneous routes, BALB/c splenocytes exhibited significantly higher proliferation index values compared to control animals (in all cases p < 0.0001) (Fig 6). In addition, N-PmpC stimulation provoked more intensive proliferation in splenic cultures from s.c.//N-PmpC EcN BGs then in cultures from conj// N-PmpC EcN BGs (p < 0.005).

### Immunization with N-PmpC EcN BGs promoted IFNγ secretion

**Fig 7:**
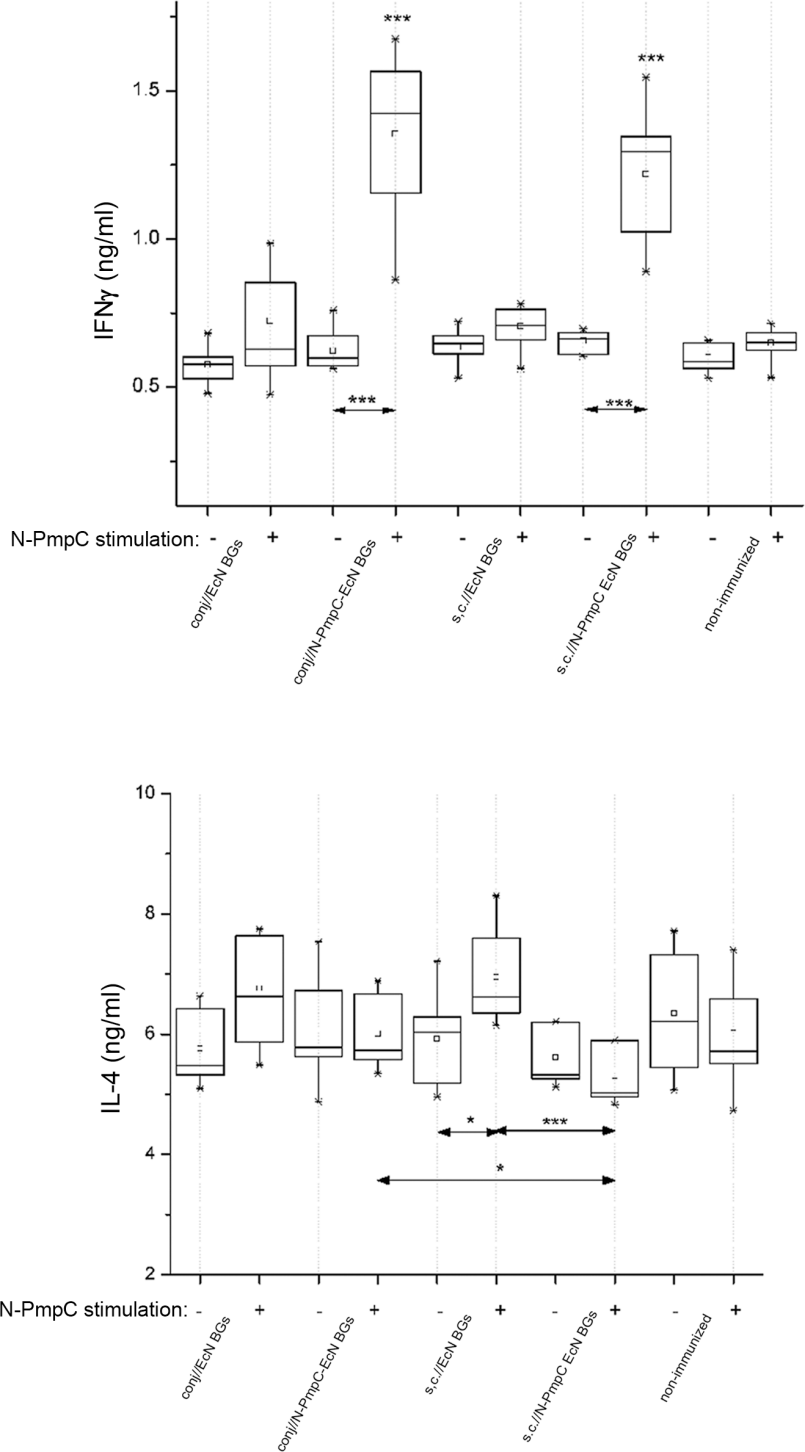
Levels of IFNγ (A) and IL-4 (B) in the supernatants from splenic cultures of N-PmpC EcN BGs-, EcN BGs- and non-immunized BALB/c mice. Mice were immunized subcutaneously (s.c.//) or via the conjunctiva (conj//). Each group consisted of 10 mice. Splenocytes were cultivated at 37°C in 5% CO_2_ for 48 h in 10% FCS/RPMI 1640/50 μM β-mercaptoethanol supplemented with 10 μg/ml N-PmpC (+) or without any additional supplementation (-). Differences in concentrations of cytokines in culture supernatants were evaluated using a 1-way repeated ANOVA (*p <0*.05*, p *<0*.005**_,_ *p < 0*.0001***). Corresponding (non-stimulated and N-PmpC-stimulated) non-immunized group cultures were used as a reference unless otherwise indicated by two head-arrow.

IFNγ and IL-4 production was evaluated in splenocyte' cultures of N-PmpC EcN BGs-immunized, EcN BG-immunized and non-immunized mice after stimulation with 10 μg/ml of N-PmpC (Fig 7). Without any stimulation, the production of tested cytokines was similar in all groups. Generally, N-PmpC stimulation did not significantly affect the production of IFNγ and IL-4 in cultures of splenocytes from control mice (immunized with EcN BGs subcutaneously and via the conjunctiva as well as non-immunized mice; p < 0.05 only of IL-4 in cultures from s.c.//EcN BGs). Upon N-PmpC stimulation, IL-4 levels revealed no differences in both N-PmpC EcN BGs immunized groups in comparison to the non-immunized control groups. However, upon N-PmpC stimulation splenocytes from s.c.//N-PmpC EcN BGs mice produced less IL-4 than splenocytes from conj//N-PmpC EcN BGs (p < 0.05) and s.c.//EcN BGs (p < 0.0001) mice. Irrespective to the immunization route, N-PmpC EcN BGs immunization resulted in increased IFNγ production in N-PmpC-stimulated splenic cultures in comparison with corresponding non-stimulated cultures and N-PmpC-stimulated cultures of splenocytes obtained from non-immunized mice (p < 0.0001 in all cases).

There were not statistically significant differences in the levels of IFNγ produced either spontaneously or upon N-PmpC stimulation by splenocytes from mice immunized with N-PmpC EcN BGs via the conjunctiva or subcutaneous routes.

### No ocular surface pathology caused by immunization with N-PmpC EcN BGs via the conjunctiva

**Fig 8:**
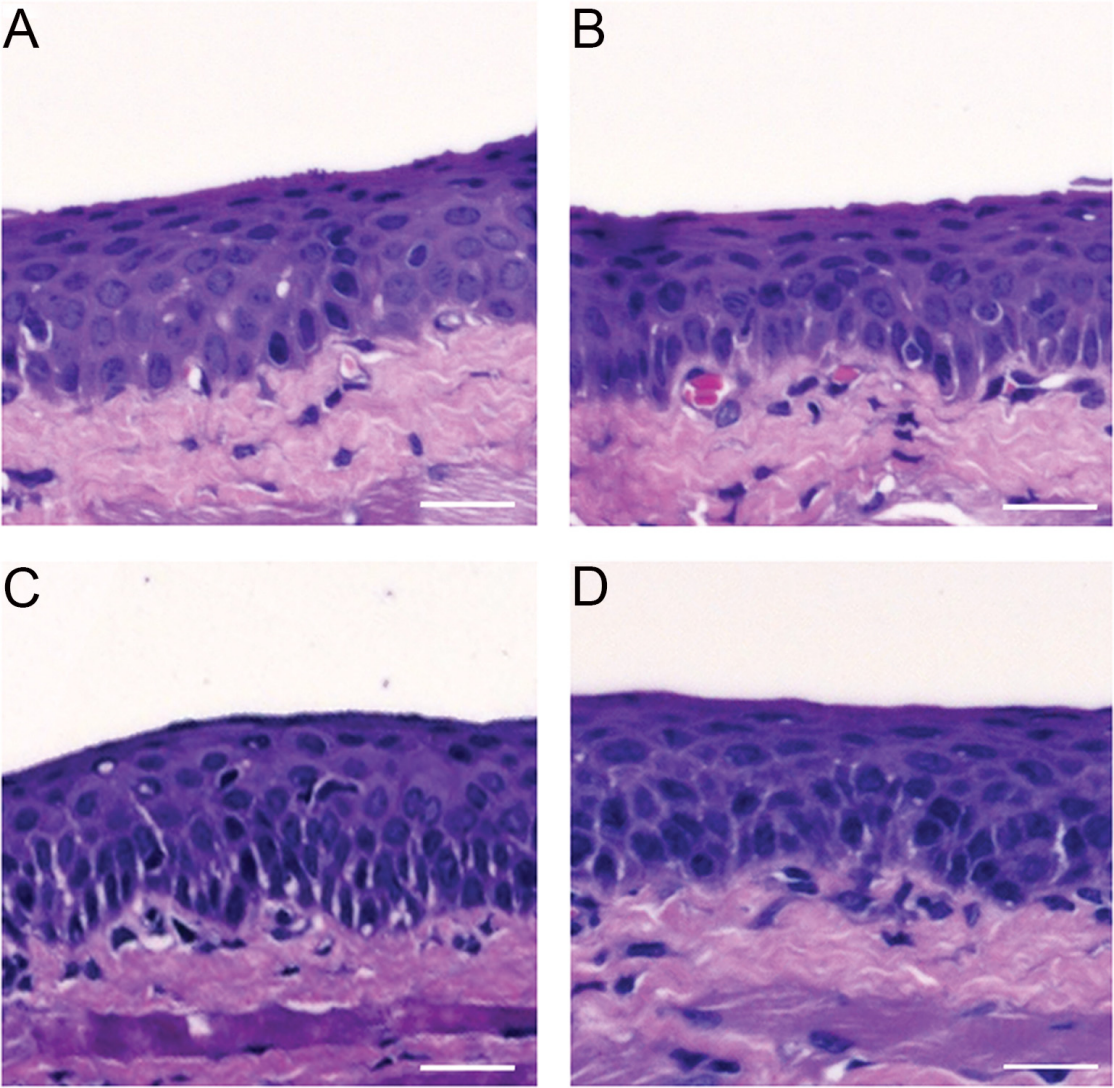
Ocular conjunctiva of N-PmpC EcN BGs-treated and control guinea pig eyes. Guinea pigs were exposed to N-PmpC EcN BGs two and 24 h. Conjunctival epithelia from both N-PmpC EcN BGs-treated and control displayed normal cell layers and morphology. No sign of tissue edema were observed in the conjunctiva studied after exposure to N-PmpC EcN BGs compared with controls. Scale bar: 10 μm. Magnification 40x.

No signs of cell infiltrates, changes in layers’ dimensions and morphology of the cells were detected at the ocular surfaces of BALB/c mice following immunization via the conjunctiva, as the eyes of non-immunized mice were visually identical to those of mice immunized via the conjunctiva. Histological analysis of the guinea pig conjunctiva confirmed the presence of normal ocular surface structures in both groups of the N-PmpC EcN BG treated eyes (Fig 8A and 8C, 2 h and 24 h, respectively) and control, non-immunized guinea pigs (Fig 8B and 8D). Conjunctival epithelia displayed a normal number of cell layers having the same dimensions as the sham-control taken samples and composed of cells with appropriate morphology. In addition, we did not observe any behavioral changes or weight loss in any mouse groups during the course of immunization.

### Partial protection against chlamydial ocular infection in the ocular guinea pig model

**Fig 9:**
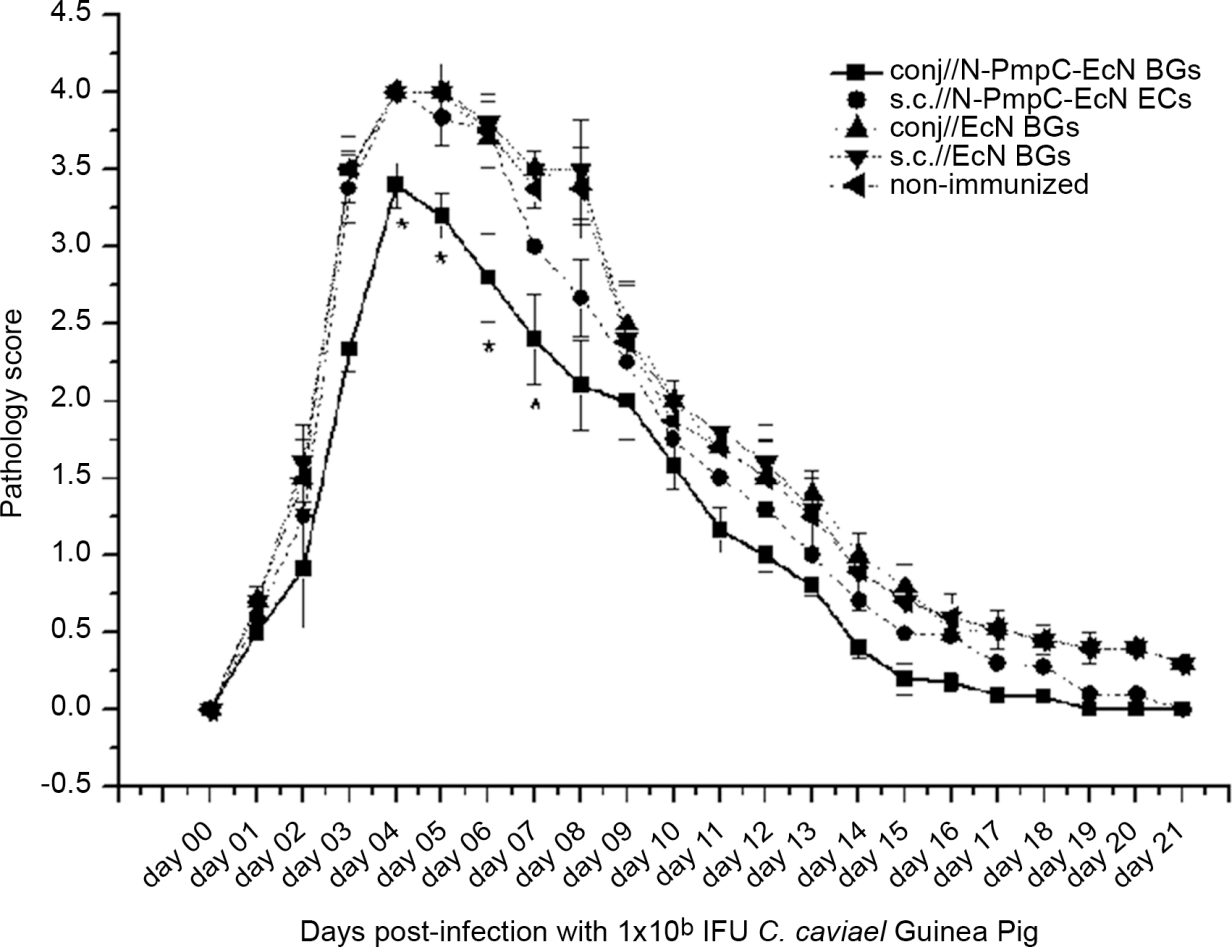
Guinea pigs were immunized with N-PmpC EcN BGs and plain EcN BGs via the conjunctiva and subcutaneously, on days 0, 14 and 28 (50 μg BGs per dose) and two weeks after the completion of the specified immunization protocol were challenged by the ocular administration of 1 x 10^6^ IFU/animal. The animals were visually assessed and scored on a daily basis. Results are presented as mean pathology score ± SD at defined time point for all animals within a group (n = 5 per group).

Non-immunized animals challenged with 1 × 10^6^ IFUs of *C*. *caviae* exhibited a more severe ocular pathological response than animals conjunctivally immunized thrice in two-week intervals with N-PmpC EcN BGs. Significantly lower pathology scores were recorded on days 4, 5, 6 and day 7 (p < 0.05) for conjunctivally immunized animals compared with non-immunized animals (Fig 9). In comparison with non-immunized animals, no statistically significant differences in pathology scores at any time point were observed for mice immunized subcutaneously with N-PmpC EcN BGs as well as for mice treated with EcN BGs via both routes.

## Discussion

Immunization via the ocular mucosa shows potential for further development to fight counter morbidity caused by ocular surface infections. As a needle-free application, topical conjunctival immunization could mitigate general vaccine safety issues [45–52] especially in developing countries by reducing the use of needles and the need for trained personnel.

Using Western blot, we first demonstrated that our antigen of interest, N-PmpC, was reliably packaged in EcN BGs and not lost during the process of *E*-lysis. Next, we demonstrated that immunization with N-PmpC EcN BGs via the conjunctiva induced N-PmpC-specific mucosal IgA immune responses at the ocular surface. Immunization with N-PmpC EcN BGs either conjunctivally or subcutaneously had no effect on serum N-PmpC-specific IgA concentrations, but mucosal IgA concentrations in tears were significantly increased in mice that were immunized via the conjunctiva compared with mice that were subcutaneously immunized. These findings are consistent with previous publications that used live-attenuated and subunit vaccines [53–55]. Local production and secretion of mucosal IgA is an important pillar of the mucosal adaptive immune response [54] and protection against infections acquired via mucosal surfaces [56]. Specific mucosal IgA neutralizes the microbes by promoting their entrapment in the mucus and may also block or sterically hinder the attachment of microbes to the epithelium [57], which could be a very significant feature for fighting/controlling ocular infections such as trachoma Ct.

Locally and systemically produced IgG protects cells by inhibiting pathogen entry, starting Fc-dependent mechanisms, and providing better antigen presentation in antigen-presenting cells. However, an IgG isotype could activate complement [57] and this process may have deleterious effects on the eye and its connecting mucosa. The process of inflammation, while important for eradicating infectious agents, is in itself harmful to the tissue [58]. Our finding that immunization via the conjunctiva does not induce serum nor tear N-PmpC-specific IgG to the same extent as that capable following subcutaneous immunization, could be interpreted as an advantage for the treatment of ocular diseases. The local increase of specific IgG may be followed by activation of certain regulatory mechanisms that limit the secretion of chlamydia-specific IgG that could be harmful to the ocular surface. The shortage of specific IgG could possibly be compensated for by the excess of locally produced mucosal IgA.

The relatively low IgG concentration during immunization with BGs via the ocular mucosa could also be explained by the fact that the antigen concentration for mucosal vaccines must be higher than the concentration for parenteral vaccines. Indeed, we were limited by the volume that could be efficiently applied onto the mouse ocular mucosa. However, it is likely that increasing the total amount of antigen administered by either increasing the amount of antigen per BG or increasing the number of N-PmpC EcN BGs per dose may lead to higher specific anti-N-PmpC IgG titers.

An analysis of cytokine production in splenic cultures upon N-PmpC stimulation revealed the significant enhancement in IFNγ levels in both conjunctively and subcutaneously N-PmpC EcN BGs-immunized mice. This finding implies that N-PmpC, in the context of EcN BGs delivery system, is capable to initiate specific IFNγ immune response which currently available data mark as indispensable to cope with chlamydial infection [17]. It is possible that some regulatory mechanisms which prevent the exacerbated immune response are also involved. This is supported by our finding that topical conjunctival application of N-PmpC EcN BGs was well tolerated in guinea pigs and mice (data not shown) with no visible pathology at the ocular surface in either animal despite the fact that the applied N-PmpC EcN BGs amount (50μg/animal) was relatively high. Further experiments will be focused on the characterization of the cytokine milieu established after BGs immunization.

The protection assay in guinea pigs revealed protection against *C*. *caviae* only in animals immunized via the conjunctiva and not in subcutaneously immunized animals. This emphasizes the importance of locally secreted N-PmpC-specific mucosal IgA.

The partial protection could be explained by the following: 1) the low dose volume we used for the conjunctival immunization, which consequently led to the low N-PmpC amount administered, and 2) the origin of N-PmpC (Ct serovar B), which probably exhibited insufficient homology with N-PmpC expressed in *C*. *caviae*.

On the other hand, partial protection observed in our experimental setting demonstrates that N-PmpC can trigger heterologous immunity. Currently we cannot draw any conclusion about the affinity and/or avidity properties of these cross-reactive antibodies and further studies are needed to address the longevity of protection. It would be important to determine if these cross-reactive antibodies were only transiently elevated or conferred lifelong (partial) protection. Others have already shown that MOMP can elicit significant heterotypic protection, in particular against pathogen burden, although not necessarily against disease status [59]. As demonstrated in mouse studies, heterologous immunity can have beneficial and harmful effects. It has been shown to confer partial protection against viral infections that are otherwise lethal [60], but can also be associated with more severe immunopathology [61].

The presented results on N-PmpC EcN BGs are consistent with earlier findings that BGs derived from different bacterial species induce specific humoral and cellular immune responses by various methods of administration *in vitro* and *in vivo* [62–68] without signs of toxicity [69]. In summary, the use of recombinant BG platform technology efficiently stimulated specific mucosal immune responses at the ocular surface and exhibited promising results as a delivery system in the development of topical conjunctival vaccines.
